# Respiratory Symptoms and Skin Sick Building Syndrome among Office Workers at University Hospital, Chiang Mai, Thailand: Associations with Indoor Air Quality, AIRMED Project

**DOI:** 10.3390/ijerph191710850

**Published:** 2022-08-31

**Authors:** Vithawat Surawattanasakul, Wachiranun Sirikul, Ratana Sapbamrer, Kampanat Wangsan, Jinjuta Panumasvivat, Pheerasak Assavanopakun, Supang Muangkaew

**Affiliations:** 1 Department of Community Medicine, Faculty of Medicine, Chiang Mai University, Chiang Mai 50200, Thailand; 2 Center of Data Analytics and Knowledge Synthesis for Health Care, Chiang Mai University, Chiang Mai 50200, Thailand; 3 Faculty of Medicine, Chiang Mai University, Chiang Mai 50200, Thailand

**Keywords:** indoor air quality, office workers, sick building syndrome

## Abstract

Sick building syndrome (SBS) is the term used to describe the medical condition in which people in a building suffer from symptoms of illnesses for no apparent reason. SBS was found to be associated with indoor air quality (IAQ) but there are a variety of determinants (buildings, in particular). Identifying and controlling factors related to SBS is crucial for improving worker health and efficiency. A cross-sectional study was conducted to investigate (1) the prevalence of respiratory symptoms and skin SBS and (2) their associations with IAQ among office workers in administrative offices in an academic medical institute. A self-reporting questionnaire assessing the worker’s characteristics, working conditions, and perception of working environments was used. The building assessment was via a walk-through survey and IAQ measurement. Of 290 office workers, 261 (90%) in 25 offices of 11 buildings took part in the survey. The highest prevalence of SBS was nasal symptoms (25.3%). We found that to reduce the risk of SBS, optimal air temperature levels in air-conditioned offices should be lower than 23 °C, with relative humidity between 60% and 70%. Lowering indoor CO_2_ levels below 700 ppm may be indicative of adequate ventilation to prevent SBS by reducing worker discomfort and indoor contaminants (e.g., formaldehyde).

## 1. Introduction

Over the past three decades, the regulated indoor ecosystem has been developed and implemented in non-industrial buildings [1]. Presently, 70 to 90% of workers work in non-industrial and indoor environments [2]. Indoor environmental quality has considerable potential to affect workers’ health, particularly sick building syndrome (SBS) [3]. SBS has become a key public health and occupational concern among office workers since most of them spend up to 90% of their working time in indoor environments [1,4,5,6].

The United States Environmental Protection Agency (U.S. EPA) introduced the concept of SBS to describe a medical condition in which people in buildings experience symptoms or feel ill for no apparent reason [7]. SBS refers to a cluster of nonspecific symptoms, including headache, fatigue, and irritation of the upper respiratory tract, nose, throat, eyes, hands, and/or facial skin [8]. It tends to increase in severity with the length of time individuals stay in a building and improve or disappear when people leave the building. SBS can occur in various workplaces, such as office buildings, universities, or hospitals [9]. According to a World Health Organization (WHO) report [10], SBS may affect approximately 30% of workers in new and renovated buildings worldwide resulting in significant loss of productivity, increased absenteeism, and increased employee turnover [1]. There are several well-established risk factors related to SBS, including personal factors and working conditions (e.g., work-related stress, psychosocial factors, and allergic conditions) [11], and building-related factors [12]. Several building-related factors, including inadequate heating, ventilation, air conditioning (HVAC) systems, humidity, noise, indoor air pollutants (IAP) (e.g., nitrogen dioxide, sulfur dioxide, ozone, particulate matter (PM), volatile organic compounds (VOCs), carbon monoxide (CO), formaldehyde), and biological agents, are all possible determinants of SBS [2,11,13,14,15,16]. Although the causes of SBS appear to be multifactorial in this complex environment, the majority of risk factors are related to indoor air quality (IAQ) [17,18].

IAQ has been a major public concern in developing countries in recent decades [12]. From previous studies in academic settings, office workers spent an average of 8.5 h in indoor environments, while the levels of IAP in office buildings were higher than levels detected outside [19,20]. IAP levels were found to be 2–4 times greater than those of outdoor air pollutants, and IAQ in an unmanaged workplace was 2–5 times worse than outside air quality [1]. According to an Occupational Safety and Health Administration (OSHA) report, 30% of workers in various industrial facilities are exposed to poor IAQ and work in substandard buildings [21,22]. Poor IAQ in buildings can be caused by a variety of factors, including the existence of local sources of pollutants, poorly planned and maintained ventilation systems, and building construction or renovation [22,23]. Typically, IAQ problems are associated with HVAC systems that have poor maintenance, resulting in insufficient ventilation and the inability to remove pollutants from the room or building [24].

CO_2_, formaldehyde, and VOCs have been proven in other studies to be major indoor air contaminants due to the lack of ventilation [22]. SBS can be caused by insufficient ventilation per person and increased levels of indoor chemical pollution [4,5]. Indoor CO_2_ concentrations could be used as a surrogate for occupant-generated pollution and indoor ventilation parameters [23,25]. However, few studies have been conducted on the assessment of IAQ and SBS among back-office workers in healthcare facilities that operate on a full-time basis, along with the continuous renovation of facilities, resulting in difficulty in IAQ monitoring and management. Identifying and controlling factors related to SBS is crucial for improving worker health and efficiency. Therefore, this study aimed to determine the prevalence of respiratory symptoms and skin SBS among office workers and their associations with IAQ in healthcare facilities.

## 2. Materials and Methods

### 2.1. Study Design and Participants

A cross-sectional study was conducted in October 2021 to investigate the prevalence of respiratory and dermal SBS and their associated factors among office workers in the academic medical institute of Chiang Mai University. This study is part of the project AIRMED, which aims to investigate the health effects of IAP on office workers at Chiang Mai University. We contacted the institution’s facility management unit to obtain a list of all offices and the number of office workers in each. We included administrative offices equipped with air conditioning and that had at least three workers. In total, there were 60 administrative offices in 11 buildings and 37 of them met the inclusion criteria. We excluded 23 offices because their workers mainly worked on non-standard office tasks. We performed simple randomization to select 25 from 37 eligible offices, including 13 from 15 support units and 11 from 22 medical departments.

**Figure 1:**
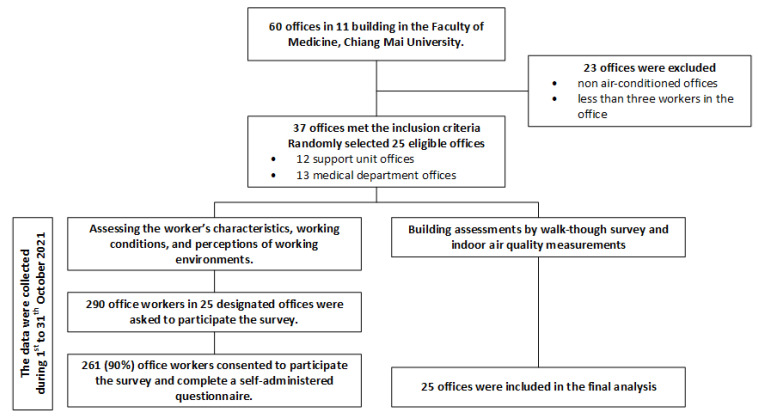
The study flow diagram.

There were 290 office workers employed in 25 designated offices. They were invited through emails, announcements, and face-to-face invitations. Participants were asked to complete a self-administered questionnaire within two weeks following the working environment assessments. There were four parts to the questionnaire, including worker characteristics, working conditions, perceptions of working environments, and SBS. Of 290 workers, 261 (90.0%) gave consent to participate in this survey and completed a self-reported questionnaire. The study flow diagram is provided in Figure 1. 

### 2.2. Questionnaire

#### 2.2.1. Worker Characteristics, Working Conditions, and Perceptions of Working Environments

The participants were asked about their age (year), gender (male/female), smoking status (non-smoker/ex-smoker/current-smoker), working experience (year), and underlying diseases as part of the worker characteristics. For the assessment of working conditions, they were asked about their work tasks, regular work (hours per week), and overtime work (hours per week) at the offices. For the perception of the working environment, they were questioned about their feelings of sensitivity to smoke and chemical agents (yes/no) in the working environment.

#### 2.2.2. Sick Building Syndromes (SBS)

The questions for assessing SBS symptoms were adapted from the standardized questionnaire, the Möljömedicin in Swedish (MM 040 NA) for workplaces [26]. It is a validated questionnaire designed for the epidemiological assessment of indoor air quality problems in workplaces and was validated in the study on office workers [26]. The questions pertained to 38 symptoms from three main groups (dermal, mucosal, and general symptoms) and perceptions of their symptoms: “Do you think that your symptoms are caused by your working environment?” (Yes/No). The frequency of each SBS symptom was indicated by five-point Likert scales during the previous three months: 3–5 days per week, 1–2 days per week, 2–3 times per month, once a month, and never. The questionnaire was translated from English into Thai and back from Thai to English by two experts who were also bilingual; one had English as a first language, and the other had Thai as a first language. Both of them are professionals in occupational health. The questionnaire’s reliability was evaluated with a group of 30 volunteers from the Faculty of Medicine, Ching Mai University (Cronbach’s alpha of 0.938).

In this study, the criteria for SBS were defined by participants having symptoms for more than 1 day per week and reporting that their symptoms were caused by their working environments. We focused on the respiratory tract and dermal symptoms of SBS and asked about 18 symptoms: (1) nasal symptoms included irritated, itching, stuffy or runny nose, and burning; (2) throat symptoms included irritated, hoarse or dry throat, burning, sore throat; (3) lower respiratory tract symptoms included cough, difficulties breathing, chest tightness; (4) dermal symptoms included symptoms dry skin, scaling or itching scalp, irritated and flushed facial skin, scaling or itching ears, hand dry, or red or itching skin. 

### 2.3. Building Assessment and Indoor Air Quality Measurements

For the assessment of the buildings, data were collected through walk-through inspections of the buildings and with questions from the research team, with the goal of surveying one set per room. Details on the type of construction and building materials, construction year of the building, type of ventilation system, the potential source of pollution, number of workers and room size, and signs of building dampness or indoor mold were noted. The inspection team consisted of multidisciplinary experts. The principal investigator was an occupational medicine physician and a safety officer. The team members were an occupational health nurse, an environmental toxicologist, and an industrial hygienist from the Ministry of Public Health to measure the environment; a research assistant was a microbiologist who collected biological parameters; and a team of engineers to inspect the building.

The IAQ was measured at a level of 75–120 cm above the room’s floor. The measurements were taken for 30 min per room, except for the PM2.5 assessment, which was performed for two hours. The number of measurement points in each room was determined by the room’s size: one measurement point per 500 m^2^, and one outdoor air quality measurement per room as recommended by Singapore Standard Council for IAQ 2021 [27]. 

#### 2.3.1. Thermal Comfort and Chemical Parameters

The Q-TRAK™ Indoor Air Quality Monitor (Model 7575, TSI Incorporated, Shoreview, MN, USA) and the VelociCalc^®^ Plus Multi-Function Ventilation Meter (Model 9565, TSI Incorporated, MN, USA) were used to measure the air temperature (AT), relative humidity (RH), air movement (AM), and chemical parameters, including CO_2_, carbon monoxide (CO) and total volatile organic compounds (TVOC). Indoor formaldehyde levels were assessed using the MIRAN SapphIRe portable interface ambient air analyzer (model MIRAN SapphIRe XL, Franklin, VA, USA). PM2.5 was measured using the DustTrak II aerosol monitor model 8530. The measurement results at each point of sampling were recorded on a field data log sheet. All instruments used were calibrated according to the manufacturers’ specifications.

#### 2.3.2. Biological Parameters

Biological parameters were examined using the NIOSH method 0800 for bioaerosol sampling (indoor air) [28] with a single-stage airborne microbial-variable impactor method with a flow rate of 28.3 L/min for four minutes. Bacteria were cultured on tryptone soya agar (TSA) media and incubated for 48 h at 35 °C. Concentrations of airborne fungi and bacteria were quantified as the number of colony-forming units per cubic meter (CFU/m^3^).

### 2.4. Statistical Analysis

The descriptive analysis was performed to describe the workers’ characteristics, working conditions, and perceptions of the working environment, as well as the results of indoor air quality measurements in 25 offices. The categorical variables were presented by the frequency with a percentage. The continuous variables were presented based on the data distributions, a mean with a standard deviation (SD) for parametric data, and a median with an interquartile range (IQR) for non-parametric data. Factors related to each SBS were determined based on the purpose of the exploratory analysis using multivariable logistic regression. We pre-defined all independent factors (both potential associated factors and confounders in a multivariable analysis) and selected these variables into the model based on their established associations with SBS from previous literature [2,11,14,15,16,29] and biological plausibility. IAQ parameters were entered into a multivariable analysis as continuous data with rescaling to detect the clinically significant level, except for formaldehyde (non-parametric data). All non-parametric data were transformed into categorical levels using quartiles to make the results from a multivariable analysis interpretable. These variables were included in the final model to obtain independent and unbiased magnitudes of association of each variable. The results from a multivariable analysis were reported as an adjusted odd ratio (aOR) with a 95% confidence interval (95% CI). All statistical analyses were performed via the statistical software STATA16 (Stata Corp. 2019, Stata Statistical Software: Release 16, Stata Corp. LLC, College Station, TX, USA). All statistical tests were two-tailed, and a *p*-value of 0.05 was considered statistically significant. The findings of the study were reported according to the STROBE (Strengthening the reporting of observational studies in Epidemiology) guidelines [30]. 

### 2.5. Ethical Considerations

This study was conducted in accordance with the Declaration of Helsinki guidelines and the protocol was approved by the Research Ethics Committee, Faculty of Medicine, Chiang Mai University, Thailand (Study Code: COM-2564-08477). This work was supported by the Faculty of Medicine, Chiang Mai University, grant no 58-2565.

## 3. Results

### 3.1. Workers’ Characteristics, Working Conditions, and Perceptions of Working Environment

Of 261 participants, the mean (±SD) age of workers was 40 (±11) years and the median (IQR) working experience was 9 (2–20) years. The majority of them were female (59.0%) and non-smokers (86.2%). Allergic rhinitis was the most commonly reported underlying disease among participants (11.9 %), whereas the majority had no underlying disease (63.2%). Most participants reported that they felt sensitive to smoke (90.8%) and chemical agents (80.3%). The mean (±SD) regular working was 37 (±8) hours per week and the median (IQR) overtime working was 2 (0–5) hours per week. Table 1 provides additional information on the working conditions and characteristics of workers who had SBS symptoms.

### 3.2. The Prevalence of SBS Symptoms

**Figure 2:**
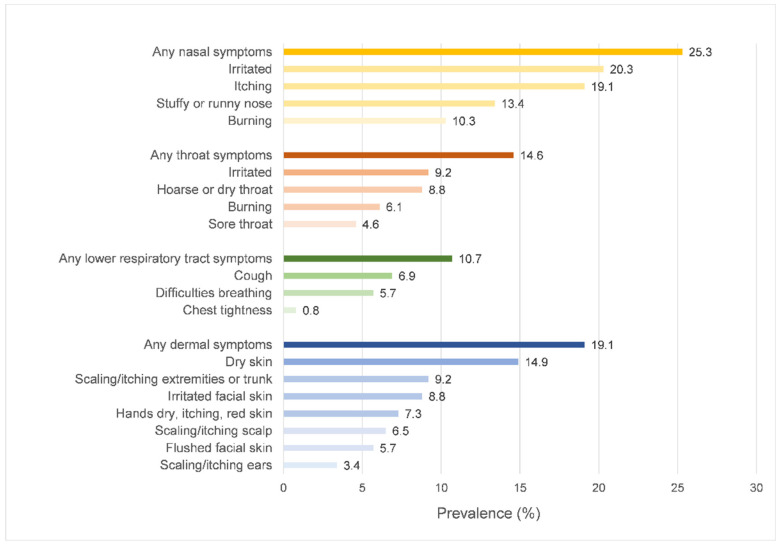
Prevalence of SBS symptoms.

From Figure 2, the highest prevalence of SBS symptoms was nasal symptoms (25.3%), followed by dermal symptoms (19.1%), throat symptoms (14.6%), and lower respiratory tract symptoms (10.7%), respectively.

### 3.3. Working Environments

Of the 25 office rooms with the IAQ parameter measurements, 4 had split unit air conditioning, 2 had centralized air conditioning, and 19 had both centralized and split unit air conditioning. There were no signs of building dampness or indoor mold growth. The average age (±SD) of office rooms was 34 (±16) years, ranging between 10 and 63 years. For thermal comfort parameters, 139 (53.3%) participants worked in the AT between 23 and 25 °C, and 113 (43.3%) worked in the AT below 23 °C. For an indoor RH, 82 (31.4%) participants worked in the recommended [27] (≥70%) and 68 (26.1%) worked in low RH (<55%). For chemical parameters, 3 offices had levels of carbon dioxide 700 ppm above outdoor, and 20 offices had formaldehyde levels over the lower limit range (>0.08 ppm) as recommended [27]. A total of 39 workers (14.9%) were exposed to high levels of indoor CO_2_, and 206 (78.9%) worked in high formaldehyde environments. Indoor CO and TVOC were not detected. The median indoor PM2.5 level in all offices was 21.0 µg/m3, while the limit levels were 37.5 µg/m^3^ [27] and 50 µg/m^3^ [29]. In addition, the average indoor air movement in all offices was below 0.30 m/s and the total viable bacterial count was below 1000 CFU/m^3^ the suggested limit level [27]. IAQ parameters of participants’ working environments are shown in Table 2. In addition, there were no significant differences in IAQ parameter levels across different air-conditioned sites, including split-type, centralized type, and both types, from the ANOVA test for parametric variables and the rank-sum test for non-parametric variables, as shown in Table S1.

### 3.4. Factors Related to SBS Symptoms

The full exploratory analysis was performed to determine the associated factors of SBS symptoms using multivariable logistic regression, including worker characteristics, underlying diseases, working conditions, perception of working environments, and IAQ parameters. The parametric variables were entered as a continuous value with rescaling to determine both clinical and statistical significance; non-parametric variables were categorized by the quartiles (formaldehyde), the limit level (PM2.5), and the median (overtime work hours per week). As shown in Table 3, the presence of allergic rhinitis was significantly associated with increases in both upper and lower respiratory tract symptoms. Increasing weekly regular working hours significantly increased the odds of nasal and lower respiratory tract symptoms. When adjusted for potential confounders and other associated factors, increasing indoor RH by 1% above the average was associated with a significant reduction in nasal (aOR 0.88, 95% CI 0.80 to 0.97; *p* = 0.007) and throat symptoms (aOR 0.87, 95% CI 0.78 to 0.96; *p* = 0.006). Changes in AT of 1 °C above the average were associated with an increase in nasal symptoms (aOR 2.63, 95% CI 1.41 to 4.90; *p* = 0.002). For chemical parameters, there were significantly increased odds of nasal (aOR 5.24, 95% CI 1.20 to 23.07; *p* = 0.029) and throat symptoms (aOR 6.45, 95% CI 1.07 to 39.01; *p* = 0.042) in the fourth quartile of formaldehyde levels (≥0.74 ppm) compared to the first quartile (<0.28 ppm). Increasing 100 ppm above the average indoor CO_2_ significantly increased the odds of nasal symptoms (aOR 1.36, 95% CI 1.03 to 1.78; *p* = 0.027), and increasing 10 CFU/m^3^ of total viable bacterial counts were also associated with nasal symptoms (aOR 1.31, 95% CI 1.03 to 1.68; *p* = 0.030).

## 4. Discussion

SBS is a group of mucosal, dermal, and general symptoms that are temporally associated with working in office-type buildings. In the present study, nasal (25.3%) and dermal SBS (19.1%) were mostly reported among office workers in the healthcare setting, followed by throat and lower respiratory tract symptoms. After adjusting for individual characteristics and working conditions, high indoor AT and low RH independently increased the odds of nasal symptoms, as well as increased the other IAQ parameters, including CO_2_, formaldehyde, and the total viable bacterial count. Low RH and high formaldehyde levels were significantly associated with increasing throat symptoms. 

In the context of the tertiary healthcare setting, our study revealed that the prevalence of respiratory and dermal SBS among office workers was relatively higher than in previous studies in different types of buildings, ranging from 11.9% to 15.9%, and 8.1% to 11.9%, for weekly respiratory and dermal symptoms, respectively [1,3,31,32,33]. Other studies in healthcare settings similarly found a greater prevalence of weekly nasal (range 8.1 to 70.5%) and dermal SBS (range 22.5 to 59.4%) than in non-healthcare settings [34,35,36]. The high prevalence of SBS among workers in healthcare settings could be influenced by multiple factors, including individual characteristics, working conditions, and building factors, especially indoor air quality.

For the thermal comfort parameters, increasing SBS with AT above 23 °C in air-conditioned buildings has been reported consistently in northern European studies [37,38,39]. However, there was an association between increasing AT, crowding, and insufficient ventilation, making it difficult to define the independent association between AT and SBS prevalence. After adjustment for these factors, our study revealed that an increase of 1 °C above the average AT (23.39 °C) independently increased nasal SBS. As recommended by countries in east and southeast Asia [27,40], the current acceptable limit was between 17 and 28 °C. Therefore, this finding suggests that maintaining an indoor AT of 23 °C or less is recommended and could reduce nasal SBS among office workers. Nevertheless, the underlying pathophysiology of this finding is uncertain and remains to be explored. 

Another thermal comfort parameter is indoor RH. The effect of indoor RH varies and depends on the outdoor climate. In Chiang Mai, Thailand, the average outdoor RH ranged from 4% in January to 94% in August 2021. The average outdoor RH declined rapidly from 93 to 76% during the study period in October. Increasing indoor RH was significantly associated with decreased nasal and lower respiratory tract symptoms in our setting, which is a tropical wet and dry country. Increasing RH was associated with reduced weekly mucosal SBS symptoms in our study. This finding is consistent with the other studies from the tropical climate zones, including Malaysia [31] and Taiwan [34]. As recommended by Singapore Standards for IAQ [27], indoor RH should be lower than 65% or 70% depending on building types, and the acceptable ranges vary between 30% and 80% according to the IAQ standards in other east and southeast Asian countries [40]. Humidification of air to achieve indoor air quality standards may prevent respiratory SBS in tropical countries and other countries [41]. However, indoor air humidification in air-conditioned buildings can cause more SBS than prevent them due to microbial contamination and chemical substances in biocides. Adding biocides to humidified water is necessary because humidifiers in the ventilation system, particularly in low RH circumstances, provide an ideal environment for microorganisms to flourish. We also found a significant association between increasing the total viable bacterial count by 10 CFU/m^3^ over the average (40 CFU/m^3^) and nasal SBS. Indoor air levels with bacterial concentrations of less than 1000 CFU/m^3^ are appropriate for preventing respiratory diseases (e.g., alveolitis, humidifier fever, and asthma) as recommended by Singapore IAQ 2021 [27] and the WHO report [42]. It might be considered a high level for SBS symptoms. The recommendation for indoor thermal comfort and biological qualities may need to be updated to enhance SBS prevention from working in a particular building.

For chemical parameters, high formaldehyde levels significantly increased the odds of upper airway SBS. Formaldehyde is commonly found in both indoor and outdoor air at low levels, often less than 0.03 ppm (range 0.01 to 0.05 ppm) for indoor environments [43,44,45,46,47]. Some individuals may have mucosal and dermal symptoms from sensory irritation at levels above 0.08 ppm and the current acceptable limit was between 0.08 and 0.1 ppm as recommended by the standard in east and southeast Asia countries [27,40]. Strikingly, 20 offices had extremely high formaldehyde levels above the recommendation, at a median (IQR) level of 0.36 (0.28–0.74) ppm. Considering all indoor sources of formaldehyde, identifying the major ones that contribute to indoor levels is difficult [48,49,50,51]. The age of the building, electronic equipment (e.g., computers and photocopiers), and other consumer items (cleansing products, insecticides, and paper products) were typical sources of indoor formaldehyde [51,52]. The extremely high formaldehyde levels in this study could be explained by the old age of the offices, poor air ventilation, and the use of high formaldehyde-emitting materials and products. Smoking in the workplace may cause temporary surge levels [53] but is not a major contributing factor in healthcare settings where smoking is prohibited. Increasing ventilation [52,54] and using low-emitting materials and products are the most effective ways to control formaldehyde concentrations. 

High CO_2_ levels were also related to increased nasal SBS. During the study period, the average outdoor CO_2_ was 700 ppm. We found that an increase of 100 ppm above the average indoor level (797.75 ± 191.36 ppm) or approximately 200 ppm over the outdoor level caused 1.38 times higher odds of nasal SBS. This finding is consistent with the previous studies in the U.S. [5,55] and Taiwan [3]. The recommended limit of indoor CO_2_ should be lower 1000 ppm [40] and the difference between indoor–outdoor levels should be under 700 ppm [27]. The association between indoor CO_2_ and SBS can be explained by the reason that high CO_2_ levels could represent an indoor environment with a low ventilation rate per occupant. This environment could increase the risk of SBS due to the discomfort caused by stuffy air and odor and the accumulation of indoor air pollutants (e.g., TVOC and formaldehyde) emitted by furniture, working equipment, and construction materials. This finding may suggest that reducing indoor CO_2_ levels should be controlled under 700 ppm in order to prevent upper respiratory SBS, although CO_2_ levels in these offices are within the normal limit range by the recommendations for office buildings. 

To the best of our knowledge, this study is the first to assess the prevalence of SBS and its associated factors among back-office workers in the healthcare sector in a developing country. Nevertheless, our findings should be interpreted with an awareness of underlying limitations. First, we conducted a cross-sectional survey from which no causal association could be directly concluded. Second, the reported SBS prevalence was a self-reported symptom and might be susceptible to recall bias. Third, the number of participants was limited, affecting the precision of the magnitude of association from a multivariable analysis. On the other hand, we are certain that the association between symptoms of SBS and independent variables was controlled for potential confounding and other determinant effects in order to estimate the unbiased magnitude of the association. Fourth, the effect estimates of confounding variables from the multivariable analysis, including worker’s characteristics, working conditions, and perception of working environments, may also be confounded by other uncontrolled confounders even though the effect estimates for the main exposures (IAQ parameters) were not confounded. In consideration of this limitation, these effect estimates should be interpreted with caution. Fifth, IAQ parameters were measured with a limited length of time, which means that the results may not be representative of the IAQ in each workplace throughout the years and may be influenced by changes in the seasons.

Our study demonstrated that IAQ was a significant determinant of respiratory and dermal SBS among office workers in a healthcare building. Thermal comfort parameters consisting of AT and RH in offices should be monitored and controlled to prevent upper respiratory SBS. We suggest that the appropriate AT levels in air-conditioned offices be lower than 23 °C to 24 °C and indoor RH be controlled between 60% and 70% as stated in the current recommendations [27]. Improving air ventilation in offices could prevent SBS from IAP, e.g., formaldehyde. CO_2_ levels could be used as the surrogate parameter for indoor air ventilation and occupant-generated pollution levels. We propose that the indoor levels of CO_2_ be less than 700 ppm or 200 ppm above outdoor levels and be reduced as far as is practical. 

In the future, we plan on measuring parameters over a longer period of time, including the other seasons, especially during the high PM season, as Chiang Mai has one of the top ten highest PM2.5 levels in the world. The population will then be expanded to include the healthcare worker group in hospitals as well as office workers in other faculties of the university. Then, in the group of rooms where we found problems with high environmental sensing values and associations with SBS, we propose adding an intervention study that aims to improve the working environment, for example by eliminating the source or improving air ventilation. After that, real-time monitoring of the operating environment for accurate post-improvement evaluations and SBS questionnaires may be collected once more to see how the prevalence of SBS has changed.

## 5. Conclusions

In the context of the tertiary healthcare setting, our study revealed that the prevalence of respiratory and dermal SBS among office workers was relatively higher than in previous studies in different types of buildings. Controlling indoor air temperatures of 23 °C or less and relative humidity between 60% and 70% is recommended to prevent upper respiratory SBS in this particular building. Increased indoor formaldehyde and CO_2_ levels were also associated with upper respiratory SBS. Eliminating formaldehyde sources should be done along with improving the indoor ventilation system. High indoor CO_2_ levels, which represent a low ventilation rate per occupant, could be indicative of a high-risk working environment. Future research should focus on developing efficient strategies for improving indoor air ventilation that could reduce indoor CO_2_ levels under 700 ppm in order to prevent respiratory SBS.

## Figures and Tables

**Table 1:** The participants’ characteristics by SBS symptoms.

Characteristics	Total (N = 261)	Nasal Symptoms (*n* = 66)	Throat Symptoms (*n* = 38)	Lower Respiratory Tract Symptoms (*n* = 28)	Dermal Symptoms (*n* = 50)
	*n* (%)	*n* (%)	*n* (%)	*n* (%)	*n* (%)
Age (year), mean ± SD	40 ± 11	38 ± 10	40 ± 12	37 ± 11	38 ± 10
Gender					
Male	107 (41.0)	17 (25.8)	13 (34.2)	8 (28.6)	14 (28.6)
Female	154 (59.0)	49 (74.2)	25 (65.8)	20 (71.4)	35 (71.4)
Working year, median (IQR)	9 (2–20)	9 (3–15)	7 (2–17)	5 (3–15)	9 (3–14)
Smoking					
Non-smoker	225 (86.2)	61 (92.4)	34 (89.5)	26 (92.9)	45 (91.9)
Ex-smoker	24 (9.2)	3 (4.6)	3 (7.9)	2 (7.1)	3 (6.1)
Current-smoker	12 (4.6)	2 (3.0)	1 (2.6)	-	1 (2.0)
**Underlying disease**					
None	165 (63.2)	38 (57.6)	20 (52.6)	11 (39.3)	26 (53.1)
Allergic rhinitis	31 (11.9)	13 (19.7)	8 (21.1)	8 (28.6)	7 (14.3)
Sinusitis	2 (0.8)	2 (3.0)	2 (5.41)	-	1 (2.0)
Asthma	1 (0.4)	1 (1.5)	1 (2.7)	-	1 (2.0)
Skin diseases	5 (1.9)	2 (3.0)	1 (2.6)	1 (3.6)	3 (6.1)
**Workers’ perception in working environments**					
Feeling sensitive to smoke	237 (90.8)	62 (93.9)	35 (92.1)	25 (89.3)	45 (91.8)
Feeling sensitive to chemical agents	190 (80.3)	53 (80.3)	28 (73.7)	22 (78.6)	37 (75.5)
**Working conditions**					
Regular working hours per week, mean ± SD	37 ± 8	40 ± 6	39 ± 4	41 ± 7	39 ± 4
Overtime working hours per week, median (IQR)	2 (0–5)	2 (0–5)	3 (0–5)	3 (0–5)	2 (1–5)
Room sizes (m^3^), *n* (%)					
≤250	89 (34.10)	26 (39.39)	10 (26.32)	10 (35.71)	16 (32.65)
251–400	60 (22.99)	17 (25.76)	12 (31.58)	6 (21.43)	14 (28.57)
401–600	34 (13.03)	5 (7.58)	6 (15.79)	4 (14.29)	5 (10.20)
601–1000	63 (24.14)	16 (24.24)	8 (21.05)	7 (25.00)	13 (26.53)
≥1000	15 (5.75)	2 (3.03)	2 (5.26)	1 (3.57)	1 (2.04)
Number of workers per room (IQR)	19 (10–28)	18 (10–28)	18 (12–28)	18 (10–29)	18 (10–31)

**Table 2:** Indoor air-quality parameters of participants’ working environments.

Parameters	Total Locations (N = 25)	
	Mean ± SD	(Min–Max)
**Thermal comfort parameters**		
Air temperature (°C)	23.39 ± 1.00	(21.40–25.60)
Relative humidity (%)	61.83 ± 5.66	(50.40–76.60)
Air movement (m/s)	0.11 ± 0.08	(0.03–0.26)
**Chemical parameters**		
Carbon dioxide (ppm)	795.75 ± 191.36	(434.00–1210.00)
Formaldehyde (ppm) ^a^	0.36 (0.28–0.74)	(0.00–2.58)
Carbon monoxide (ppm)	ND	ND
TVOC (ppb)	ND	ND
**Particulate matter**		
PM2.5 (µg/m^3^)	21.0 (13.0–29.0)	(3.0–65.0)
**Biological parameters**		
Total viable bacterial count (CFU/m^3^)	40 ± 20	(6–78)

^a^ Non-parametric data are presented using median (IQR); abbreviation: CFU/m^3^, colony-forming units per cubic meter; ND, not detected; ppb, parts per billion; ppm, parts per million; TVOC, total volatile organic compounds.

**Table 3:** The associated factors of SBS by a multivariable logistic regression model.

Variables	Nasal Symptoms Model	*p*-Value	Throat Symptoms Model	*p*-Value	Lower Respiratory Tract Symptoms Model	*p*-Value	Dermal Symptoms Model	*p*-Value
	aOR (95% CI)		aOR (95% CI)		aOR (95% CI)		aOR (95% CI)	
**Characteristics**								
Age (years)	0.98 (0.94 to 1.03)	0.401	1.03 (0.98 to 1.08)	0.240	1.00 (0.94 to 1.06)	0.930	1.00 (0.95 to 1.04)	0.846
Gender								
Male	Ref.		Ref.		Ref.		Ref.	
Female	**2.38 (1.10 to 5.14)**	**0.028**	1.14 (0.50 to 2.64)	0.746	1.22 (0.42 to 3.59)	0.715	1.81 (0.83 to 3.98)	0.134
Working year	1.00 (0.96 to 1.05)	0.866	0.97 (0.92 to 1.02)	0.184	0.97 (0.91 to 1.04)	0.383	0.99 (0.94 to 1.03)	0.672
Smoking								
Non-smokers	Ref.		Ref.		Ref.		Ref.	
Current/Ex-smokers	0.98 (0.43 to 6.76)	0.987	0.78 (0.08 to 7.44)	0.832	1.32 (0.23 to 7.69)	0.753	0.61 (0.06 to 5.78)	0.669
**Underlying disease**								
Allergic rhinitis	**2.59 (1.00 to 6.68)**	**0.050**	**2.88 (1.06 to 7.85)**	**0.038**	**5.03 (1.65 to 15.38)**	**0.005**	Not included	
Skin diseases	Not included		Not included		Not included		6.66 (0.93 to 47.60)	0.059
**Workers’ perception of working environments**								
Feeling sensitive to smoke	0.93 (0.24 to 3.60)	0.920	1.20 (0.28 to 5.23)	0.804	0.48 (0.10 to 2.43)	0.376	0.76 (0.21 to 2.81)	0.686
Feeling sensitive to chemical agents	1.91 (0.83 to 4.43)	0.134	0.89 (0.36 to 2.23)	0.808	1.52 (0.47 to 4.93)	0.485	1.21 (0.52 to 2.78)	0.660
**Working conditions**								
Regular working hours per week	**1.08 (1.02 to 1.16)**	**0.013**	1.04 (0.98 to 1.10)	0.227	**1.12 (1.03 to 1.22)**	**0.008**	1.05 (0.99 to 1.10)	0.130
Overtime working > 2 h per week	0.95 (0.88 to 1.02)	0.174	1.01 (0.95 to 1.07)	0.798	0.94 (0.83 to 1.06)	0.266	0.93 (0.85 to 1.03)	0.168
Room sizes (m^3^), increasing 100 m^3^	1.14 (0.99 to 1.32)	0.067	1.11 (0.95 to 1.30)	0.173	1.07 (0.89 to 1.29)	0.456	1.01 (0.88 to 1.16)	0.867
**Thermal comfort parameters**								
Air temperature (°C), increasing 1 °C	**2.63 (1.41 to 4.90)**	**0.002**	1.86 (0.91 to 3.83)	0.088	1.87 (0.82 to 4.29)	0.139	1.32 (0.72 to 2.40)	0.371
Relative humidity (%), increasing 1%	**0.88 (0.80 to 0.97)**	**0.007**	**0.87 (0.78 to 0.96)**	**0.006**	0.98 (0.87 to 1.10)	0.754	0.94 (0.86 to 1.02)	0.144
**Chemical parameters**								
Carbon dioxide(ppm), increasing 100 ppm	**1.36 (1.03 to 1.78)**	**0.027**	1.16 (0.84 to 1.60)	0.353	1.26 (0.89 to 1.80)	0.198	1.22 (0.93 to 1.61)	0.158
Formaldehyde (ppm) Q1, <0.28	Ref.		Ref.		Ref.		Ref.	
Q2, 0.28 to 0.35	0.90 (0.16 to 2.83)	0.853	0.32 (0.08 to 1.39)	0.128	1.19 (0.25 to 5.71)	0.824	0.68 (0.21 to 2.18)	0.521
Q3, 0.36 to 0.73	**3.88 (1.38 to 11.09)**	**0.010**	1.44 (0.40 to 5.18)	0.577	1.71 (0.40 to 7.30)	0.472	1.45 (0.52 to 4.03)	0.478
Q4, ≥0.74	**5.24 (1.20 to 23.07)**	**0.029**	**6.45 (1.07 to 39.01)**	**0.042**	1.31 (0.19 to 8.95)	0.783	1.89 (0.46 to 7.76)	0.376
PM2.5 ≥ 50 µg/m3	0.88 (0.16 to 4.73)	0.877	0.43 (0.06 to 2.90)	0.384	1.98(0.25 to 16.10)	0.524	0.63 (0.10 to 4.00)	0.621
**Biological parameters**								
Total viable bacterial count (CFU/m^3^), increasing 10 CFU/m^3^	**1.31 (1.03 to 1.68)**	**0.030**	1.23 (0.89 to 1.69)	0.204	0.96 (0.70 to 1.30)	0.784	1.12 (0.88 to 1.43)	0.346

Bold aOR and *p*-value indicate a statistically significant association by *p*-value ≤ 0.050. Abbreviation: aOR, adjusted odds ratio; CFU/m^3^, colony-forming units per cubic meter; ppb, parts per billion; ppm, parts per million; Ref., reference category for multinomial variables; Q, quartile.

## Data Availability

The data presented in this study are available upon request from the correspondent author.

## Supplementary Materials

The following supporting information can be downloaded at: https://www.mdpi.com/article/10.3390/ijerph191710850/s1, Table S1: The comparison of IAQ parameter levels by types of air-conditioning (split-type, centralized type, and both types).
