# Supplementary material for: Respiratory Symptoms and Skin Sick Building Syndrome among Office Workers at University Hospital, Chiang Mai, Thailand: Associations with Indoor Air Quality, AIRMED Project
Source: Int J Environ Res Public Health. 2022 Aug 31;19(17):10850. doi: 10.3390/ijerph191710850 (PMC9518424; doi:10.3390/ijerph191710850)
Supplement: Supplementary file 1 [file ijerph-19-10850-s001.zip › ijerph-1853574-supplementary.pdf]

Table S1. The comparison of IAQ parameter levels by types of air-conditioning (split-type, centralized type, and both types)

Parameters	F-statistic	*p*-value
**Thermal comfort parameters**		
Air temperature)°C(	1.102	0.350
Relative humidity)%(	1.403	0.267
Air movement)m/s(	1.017	0.378
**Chemical parameters**		
Carbon dioxide)ppm(	0.468	0.633
Formaldehyde)ppm([a]	3.449	0.178
Carbon monoxide)ppm(	N/A	N/A
TVOC)ppb(	N/A	N/A
**Particulate matter**		
PM2.5)µg/m^3(	2.265	0.128
**Biological parameters**		
Total viable bacterial count)CFU/m^3(	0.476	0.628

p-value was obtained by ANOVA test, except [a] non-parametric variable, the statistical analysis was performed using Rank-sum test (X^2 statistic).
